# Supplementary material for: Asymmetric Synthesis of Quaternary Hydantoins via a Palladium-Catalyzed Aza-Heck Cyclization
Source: J Am Chem Soc. 2025 Nov 14;147(49):44692–8. doi: 10.1021/jacs.5c16022 (PMC12703750; doi:10.1021/jacs.5c16022)
Supplement: Supplementary file 2 [file ja5c16022_si_002.zip › All NMR FID Files/6/6_ProtonNMR/1/pdata/1/email_EM04-264-1-flush_1_1.pdf]

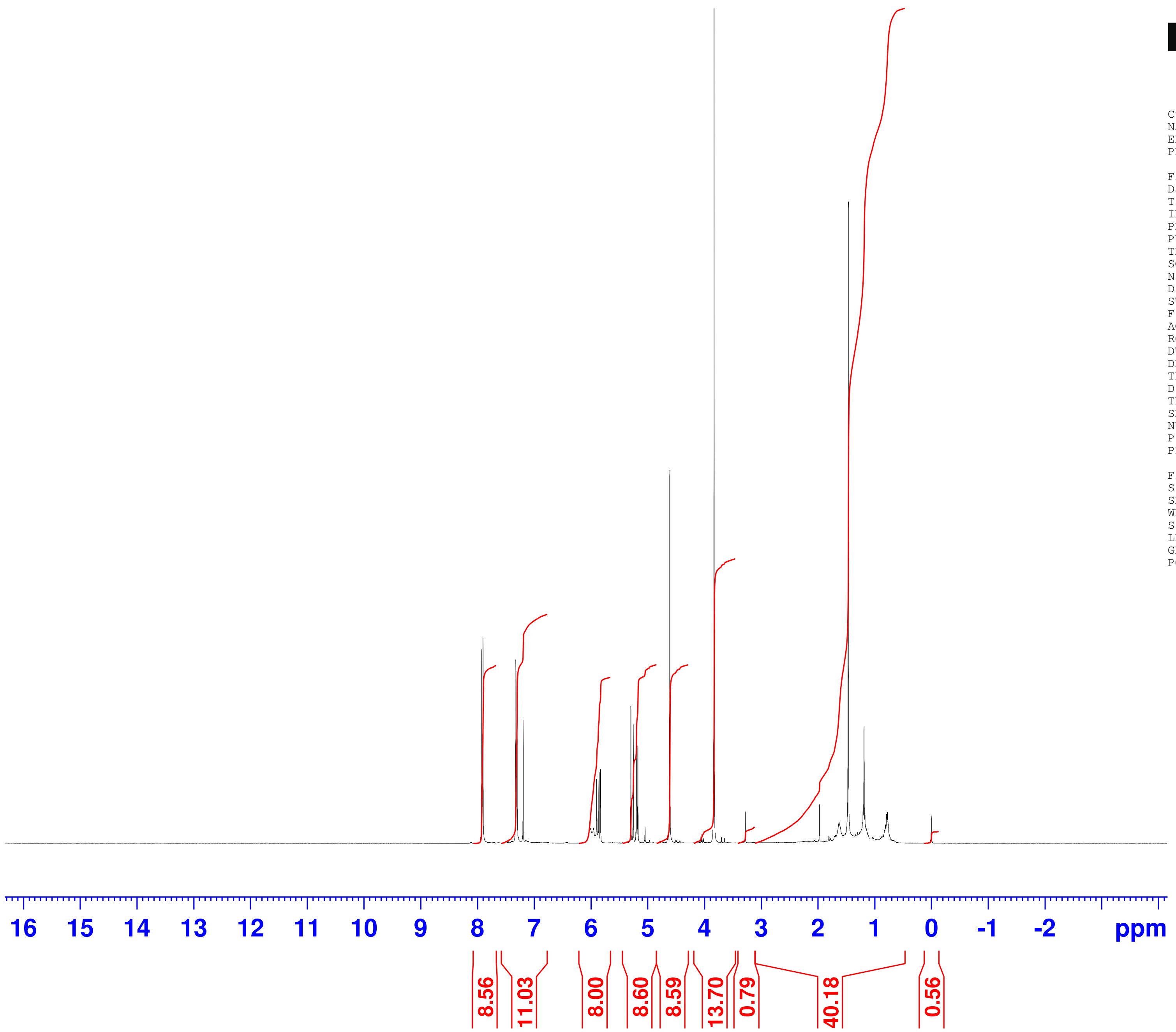

BRUKER

Current Data Parameters

NAME	EM04-264-1-flush	
EXPNO	1	
PROCNO	1	

F2 - Acquisition Parameters

Date_	20231109	
Time	17.04	h
INSTRUM	Avance Neo 400	U of DE Brown Lab
PROBHD	Z104450_0412 (	
PULPROG	zg	
TD	65536	
SOLVENT	CDCl3	
NS	16	
DS	2	
SWH	8196.721	Hz
FIDRES	0.250144	Hz
AQ	3.9976959	sec
RG	101	
DW	61.000	usec
DE	13.33	usec
TE	298.0	K
D1	10.00000000	sec
TD0	1	
SFO1	400.1324708	MHz
NUC1	1H	
P1	15.00	usec
PLW1	8.31639957	W

F2 - Processing parameters

SI	65536	
SF	400.1300360	MHz
WDW	EM	
SSB	0	
LB	0.30	Hz
GB	0	
PC	1.00	
